# On the Edge Between Digital and Physical: Materials to Enhance Creativity in Children. An Application to Atypical Development

**DOI:** 10.3389/fpsyg.2020.00755

**Published:** 2020-05-08

**Authors:** Michela Ponticorvo, Luigia Simona Sica, Angelo Rega, Orazio Miglino

**Affiliations:** ^1^Natural and Artificial Cognition Laboratory, Department of Humanistic Studies, University of Naples “Federico II”, Naples, Italy; ^2^Institute for Research, Training and Information on Disabilities (IRFID), Neapolisanit, Ottaviano, Italy; ^3^Institute of Cognitive Sciences and Technologies, National Research Council, Rome, Italy

**Keywords:** educational materials, creativity, game-based learning, video-modeling, Autis Spectrum Disorder

## Abstract

The 4 P’s creativity model (person, process, press, and product) underlines how creativity is strongly connected with the materials employed to conceive and realize a creative outcome. As a multiform construct, it invites a wide variety of approaches to the study of it. One of the most promising ways to address this issue is to connect it with cognitive development and related educational pathways, as creativity can be enhanced and stimulated in every child, leading to an improvement both at personal and societal level. Even if creativity is recognized and highly valued, there is still a lack of methods which can stimulate creativity in an effective way. Useful hints may come from the outstanding contributions of Piaget and Montessori who underlined that interaction with the physical world is a fundamental building block for cognitive development. In this paper, starting from these fixed points, we describe some creativity enhancing methods for children which give importance to the edge between digital and physical materials. Digital materials open new ways to the use and integration of physical materials with hybrid platforms which can be used in educational contexts. Together with this perspective we provide a description of the application of these methodologies to enhance creativity in children with Autistic Spectrum Disorder.

## Introduction: Where Creativity Is

A general definition of creativity describes it as the ability to generate ideas, insights and solutions that are original, flexible (Amabile, 1996; Sternberg and Lubart, 1996) and effective (Runco and Jaeger, 2012). A vast body of research has been conducted in this field from different points of view (psychological, pedagogical, educational, etc.). In brief, creativity can be understood as the combination of several factors (Treffinger et al., 1983; Houtz and Krug, 1995a, b) of both a cognitive (primarily related to divergent thinking) and an emotional type (primarily related to creative personality). On the cognitive side, there is a general convergence on the notion that creative outputs result from cognitive flexibility (flexible and divergent ways of thinking) and cognitive persistence (persistent and systematic way of thinking) (see Dietrich and Kanso, 2010). On the emotional side, Williams (1994) explored the emotional-divergent aspect of creativity, identifying the main characteristics of personality as: (1) curiosity (the capacity to investigate elements and ideas, finding new and not always direct and obvious connections); (2) complexity (the tendency to look for new alternatives and solutions to problems, to restore order out of chaos); (3) imagination (the ability to visualize mental images); (4) risk-taking (the inclination to act under unstructured conditions and to defend one’s own ideas).

**FIGURE 1:**
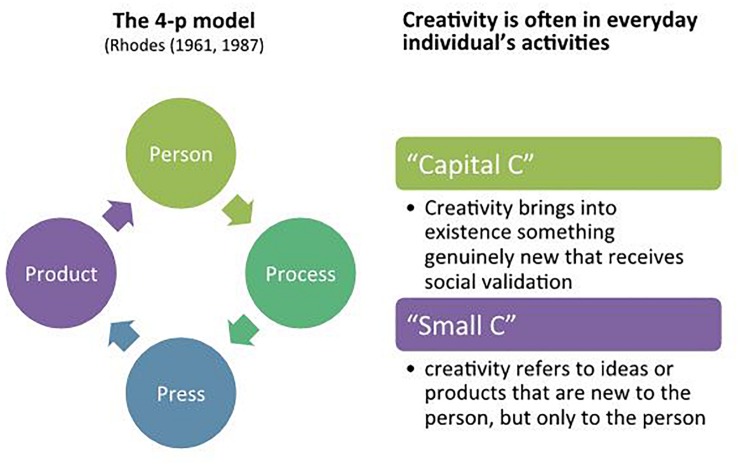
Two creativity models.

Creativity can be also seen as the result of interaction between the individual, the domain, and the field. For instance, Rhodes (1961, 1987) developed the 4 P’s model (Figure 1), which places creativity at the interplay of four distinct strands, i.e., *process* (the different stages of a creative activity), *person* (the characteristics of individuals), *press* (the qualities of the environment where creativity happens), and *product* (the tangible or intangible outcomes of the creative process). Rhodes’ classification has become a major framework for the holistic exploration of creativity.

However, creativity is not only the production of something new for the entire society (like arts): creativity is often found in an individual’s everyday activities. In this sense, literature defines two types of creativity: *Creativity* and *creativity*. “Capital C” creativity brings into existence something genuinely new that receives social validation (enhances culture) and is related to an accomplishment that consists of a clear-cut, eminent creative contribution. “Small C” creativity refers to ideas or products that are new to the person, but only to the person; it is therefore more focused on everyday activities, such as those creative actions in which everyone can be involved every day (Figure 1). Kaufman and Beghetto (2009) add another 2 Cs to their model, including a new category, a “little-c” for the little-c category, mini-c (Beghetto and Kaufman, 2007), which is connected with the learning process. Mini-c is defined as the novel and personally meaningful interpretation of experiences, actions, and events (Beghetto and Kaufman, 2007). Together with Mini-c they introduce Pro-c, the developmental and effortful progression beyond little-c, not reaching Big-C status, on professional-level expertise in creative areas. The 5 A’s framework (Glãveanu, 2013) goes beyond the 4 P’s model to give a new perspective on creativity: it introduces actor, action, artifact, audience, and affordances.

Considering these contributions, creativity is a precious resource for the positive psychological development of all individuals (with normative and non-normative developmental trajectories). In these terms, mainly considering the four P’s model and the Small C description, as shown in Figure 1, we deal with it.

In the present paper we aim at delineating some methods that can be applied to stimulate creativity in children with typical and atypical developmental trajectories, employing both digital and physical materials and joining the notable advantages that these kinds of materials can offer. After a description of the connection between creativity and interaction with physical materials, we describe an application of this method to a concrete case of atypical development. In particular we report in section “Fostering emotion recognition to stimulate creativity by technology in children with Autistic Spectrum Disorder (ASD)” an example of how to stimulate creativity by promoting emotion recognition with digital and physical materials in children with Autistic Spectrum Disorder (ASD).

### Creativity From a Developmental Point of View

From what we said, it is clear that creativity can also be seen as a cognitive resource along the “life-span,” starting from childhood. Indeed, children tend to exhibit a natural disposition toward creativity and expression; fluency (the ability to generate multiple ideas) and flexibility develop across adolescence with distinct trajectories for divergent thinking and insight (Kleibeuker et al., 2013), explorative thinking characterizes middle adolescence (Johnson and Wilbrecht, 2011). Moreover children can be sensitive to creativity outcomes from 3 years of age, which is quite early (Di Dio et al., 2007), and this sensitivity can enable them to develop their creative side. In children with atypical developmental trajectories, creativity can offer support for adaptive processes (Hetzroni et al., 2019). If not stimulated, creativity can diminish and follow a negative bending. For instance, creativity diminishes by 40% between the ages of five and seven. This is due to the beginning of formal schooling with its educational rules which may inhibit commitment to creative thinking and behaviors (Amabile, 1996; McCormick and Plugge, 1997). New research has also led to a new understanding of the capacity for positive change and creative expression in the second half of life (Cohen, 2006). In general terms, psychological literature has highlighted that creativity can be interpreted as an individual resource, as a potent predictor of social problem-solving and adjustment (Ogoemeka, 2011). In other words, creative individuals are remarkable for their ability to adapt to almost any situation and exploit whatever possible to reach their goals (Csikszentmihalyi, 1996).

The paths to support individual development do not always consider creativity as a useful resource for well-being, despite research providing evidence to this effect. The role of creativity as a resource for individual well-being was identified: creativity and learning are strictly connected not only during childhood but also during young adulthood and adulthood (see Gajda et al., 2017); long-term participation in creative activities has benefits for middle-aged and older people as they may improve their adaptation to later life (Adams-Price et al., 2018).

Even if we consider childhood, which is probably the most studied phase of development for supporting creativity in learning contexts, there is still a lack of methods for stimulating creativity in an effective way. In this paper we describe some methods for enhancing creativity in children that give importance to the fertile edge between digital and physical materials.

### Creativity: How to Stimulate It

The crucial added element in Rhode’s vision is the “press” or the environment. This dimension focuses on the characteristics of the environment (social, cultural, and political; Simonton, 1999) as crucial factors for supporting/hindering creativity. In addition, Csikszentmihalyi (1996) highlighted some environmental features which may foster creativity, including training, expectations, resources, recognition, and some factors which may hinder creativity, like time pressure, evaluation, lack of respect, and competition.

For Harris and de Bruin (2018) creativity is “an essential aspect of teaching and learning that is influencing worldwide educational policy and teacher practice, shaping the possibilities of 21st-century learners”^1^. Unfortunately, approaches are not always coordinated with each other and often have characteristics of extemporaneousness and occasionality. As recent work shows, there is a strong will to help teachers in enhancing creativity without the need for special programs or training, encouraging it during teachers’ regular work (Beghetto, 2013) or their work associated with the common core (Beghetto et al., 2014; Giglio and Cruz-Ortiz, 2015). In this sense, Karwowski’s et al. (2015) words on the relationship between creativity and education are enlightening:

“Since the beginning of creativity theory, the educational side of creativity has been at the heart of scholars’ thinking and research (p. 165). However, to help teachers stimulate creativity effectively, a better understanding of mechanisms underlying creativity is necessary (p.166)”.

In the field of education and pedagogy, creativity can be defined as “purposive imaginative activity generating outcomes that are original and valuable in relation to the learner” (Cremin et al., 2013; see also Craft et al., 2014).

We believe also that a thorough reflection on the tools used in the educational dialogue to stimulate the creative process is even more necessary because it ties together relational processes, cognitive processes and the instrument’s own characteristics (in terms of potential and risks). Physical objects, digital tools, and “materials” in general thus become our specific object of investigation. Moreover, thanks to the massive entrance of technological devices in all aspects of our everyday life, it is important that the concept of creativity is rethought considering these elements.

## Creativity and Interaction With the Physical World During Individual Psycho-Social Development

In order to better understand how technology can be connected to creativity in a developmental perspective, it is useful to go back to the contribution of relevant authors in the field who underline that the interaction with the physical world is fundamental in structuring cognitive processes.

Children explore the world around them by relying on their sensorimotor functions: in their infancy (from a few months to when they enter kindergarten) the hands are the main channel for acquiring knowledge. Children point, handle, touch, taste, smell, and manipulate while understanding an object’s features and functions.

As time goes by, adults do not spend the same amount of time in pointing, reaching, touching and manipulating, but the manipulative acts in the physical world become internal processes, cognitive functions and neural structures: an action is no more run in the physical world, but is simulated in the virtual space represented by the mind (Smith and Gasser, 2005).

Even if these functions become virtual, the use of hands and of the body as a whole remains fundamental: the physical world is mirrored on the cognitive side and the dynamic interaction at the edge of physical and cognitive is a resource for various cognitive processes, including creativity.

Piaget (1952, 1964), whose theorization analyzed in detail how children develop their cognition, recognizes a fundamental role for the interaction with the physical universe in shaping development at a cognitive level. In his well-known definitions of assimilation and adaptation, what comes from the external world is important also in terms of physical interaction. In this perspective, cognitive processes emerge with doing and interacting.

Vygotsky (1978), on the other hand, underlined the role of context in shaping learning and development, because, in his opinion, cognitive development is the result of an interaction between the child and the social context he/she is immersed in. The cognitive functions of a growing child are built from what happens in their social interactions (consider for example language and thought). Even if for this author the physical world is shaded, it is anyway out of doubt that social interactions happen and are mediated by physical interactions, especially during childhood.

Bruner (1961), in his theory, took some elements from Piaget, i.e., the learner active involvement and from Vygotsky, i.e., the importance of social context. Then he formulated an approach to learning cognitive development which is defined as cultural. Bruner’s learning theory states that it is a complex activity where we can recognize three underlying interacting processes: (1) acquisition of information, (2) transformation of information in a new form that allows problem solving and (3) checking the efficacy of this transformation.

Bruner gives central importance to culture and to the active participation of children for their cognitive development. Bruner in fact stated that “the active participation in the learning process by the child might result in the following hypothesized benefits: an increase in intellectual potency so as to make the acquired information more readily viable in problem solving, the action of the learning activities in terms of the intrinsic reward of discovery itself (as contrasted with the drive-reduction model of learning), learning the heuristics of discovery, and making material more readily accessible in memory” (Bruner, 1961, p. 21).

Starting from Bruner, other authors in the cognitive field have stressed this issue; in their contribution Papert (1980) and Jonassen (1994) underline the active role of the people who get to understand their experience by exploiting cultural tools in a context. In this approach, which has been named in constructionism, “meaningful learning” (Jonassen et al., 2008) and “discovery learning” (Papert and Harel, 1991) are important conceptual contributions and cannot be neglected when reflecting on creativity and technology.

More recently, the perspective of embodied and situated cognition (Clark, 1998; Shapiro, 2019) puts the concept of action at the center together with physical interaction. In embodied cognition theory, the organism with its sensory and motor apparatus interacts with the external environment and structures its cognitive processes through this interaction. This means that the body, by means of its continuous exchange with the world, both physical and social, determines how cognition develops. The motor and the perceptual apparatus allow the constant flow of information between the internal and external side that sediment in the cognitive processes.

For the situated learning theories (Clancey, 1995) the theoretical core is that the subject (who knows) cannot be considered as different and separated from the object (what is known). Moreover this process does not happen in a vacuum, but in a context defined by social constraints (Rambusch, 2006). Along with this approach, it is important to underline that the body is a relevant medium of exchange between person and context. This argument dates back to Piaget.

For Piaget and his seminal theory, the body is the first instrument, in terms of time and of importance, to get to know the world. Indeed in his theory, the sensorimotor stage is the first phase in which children both with typical and atypical development exploit his/her body to explore the environment. More recently the contribution by Galperin (1969, 1989) supports the idea that body is fundamental for mind: the mental object-oriented activity derives from the object-oriented activity which is run in the physical world, at the beginning. In brief, the physical manipulation of tangible objects is the starting point of what is later internalized and becomes human thought. Galperin’s approach has been considered by the later contribution of Rambusch and Ziemke (2005) as a bridge, a connection between situated learning and embodied cognition theories.

Using Rambush and Ziemke words, we can say that “the embodied cognition is in many aspects a very social process, and that embodied social process such as mimicry and imitation are significant for social relations as they help people connect, making it possible for them to communicate and to understand each other.” The work of Roth (2002) falls perfectly in this trace. In fact he showed that gestures are not only a reflection of what has been learnt but also contribute to new acquisition, because they have the function to communicate to the external world together with helping to make things clearer and more understandable for the speaker itself.

In the field of education science, relevant approaches have been affirmed along the years which hold, at their core, the importance of child-active involvement. Let us consider the Montessori (2013) approach, which suggests using methodologies where children are at the center, acting with special materials that stimulate child senses, for example, smelling jars, the broad stair and the pink tower. Children play with these materials in a way that promote their independence in learning and their problem-solving ability, together with peer cooperation. This leads to an active education methodology that can be fruitfully applied in the acquisition of cognitive and social skills, including creativity.

In this approach, the hands play a fundamental role. In Montessori’s (1995) words, hands are instruments of intelligence, they become an extension of thought and can become a vehicle to stimulate creativity, confirming what has been said about Embodied Cognition. In our opinion, the described contributions underlying the importance of the body in exploring and acquiring knowledge about the word have important implications in the field of education and in the development of creativity. As underlined by Stanciu (2015), the core ideas of embodied cognition can have a notable effect on the discussion on creativity in cognitive science, especially in the domain of everyday creativity, the little-c, but also with the forms which result in culturally relevant, highly regarded products and artifacts (e.g., Kaufman and Beghetto, 2009).

We therefore believe that creativity can be stimulated keeping in mind the contribution from Embodied Cognition theory, allowing to exploit the process in which the body and the environment can shape creativity. Creativity in the Embodied Cognition framework can help us to understand the impact that physical and body activities have on creative thinking, also on typical and atypical development. This means that techniques based on Embodied Cognition can foster a creative output.

In the next section we will introduce some tools and methods used to enhance creativity in children, based on the described theoretical framework. Moreover we will introduce, in section “Fostering emotion recognition to stimulate creativity by technology in children with Autistic Spectrum Disorder (ASD)”, a possible application of the tools and methods used to stimulate creativity in children with Autistic Spectrum Disorder as reported in section “Methods to enhance creativity in children: playing implies learning in a creative way”.

## Methods to Enhance Creativity in Children: Playing Implies Learning in a Creative Way

“We can identify creative processes in children at the very earliest ages, especially in their play. A child who sits astride a stick and pretends to be riding a horse; a little girl who plays with a doll and imagines she is its mother; a boy who in his games becomes a pirate, a soldier, or a sailor, all these children at play represent examples of the most authentic, truest creativity. (…) A child’s play is not simply a reproduction of what he has experienced, but a creative reworking of the impressions he has acquired” (Vygotsky, 1967; Engl. Transl., 2004, p. 11). We can start our discourse from this relationship between creativity and play identified by Vygotsky. That is also our argument: stimulating play can stimulate learning, and both imply creative processes. More specifically, to learn by exploring reality (objects, rules and roles, questions, problems) is a process involving curiosity, flexibility, divergent thinking, and risk-taking, all of which are creative processes.

During the entire developmental arc, from birth to elder age, playing a game stimulates the player to start a challenge, either cognitive (card games, board games, role games) or physical (competition in sport, dancing, fine manipulative activities). This challenge leads the player to learn something (or to exercise already acquired abilities), and so playing a game can be a good approach to transferring some knowledge or skill at every age (Dell’Aquila et al., 2016).

What we have said about games in general is also valid for digital games. These have become very widespread nowadays and are often used in a game-based learning approach. Indeed Tobias et al. (2014) in their fundamental review on this issue have shown that using video and computer games is an effective way to enhance the cognitive processes underlying learning. This effectiveness is strengthened if game design and instructional design are integrated to exploit at the maximum level the motivating features of games. In fact, games are exceptionally good at involving people and increasing their motivation to solve problems, promoting learning (Kapp, 2012).

Along with game-based learning, technology-enhanced learning (TEL), technology-enabled learning or technology-enhanced education (TEE) are a wide ensemble of educational methodologies based on digital technologies that give importance to interactivity in the learning process, active experience and collaborative knowledge building (Goodyear and Retalis, 2010).

TEL includes Serious games, Augmented Reality, Educational Robotics and Multiplayer Virtual Worlds, educational technologies, e-learning, technology-enhanced classrooms, etc. TEL can be used to design and implement different kinds of technology-supported learning that is strongly activity-based. In fact TEL has some relevant features that can be fruitfully employed to stimulate cognitive processes: interactivity, collaboration, communication, personalization, etc.

TEL allows children to have more control of their own cognitive processes, to build up knowledge, and become part of the teaching process, both individually and as a group. Indeed collaboration between peers becomes relevant and can be very well supported in a TEL context. This means that TEL can lead to really engaging learning experiences where different tools can be used to build personalized learning pathways. As children today are more and more exposed to and attracted by technologies, TEL can exploit this attractive power.

The introduction of TEL can effectively exploit students’ potential in a tailored scenario, and motivate children through creativity and customized resources. On the educator side, this means that they modify their pedagogy and educational models to make education an active process.

**FIGURE 2:**
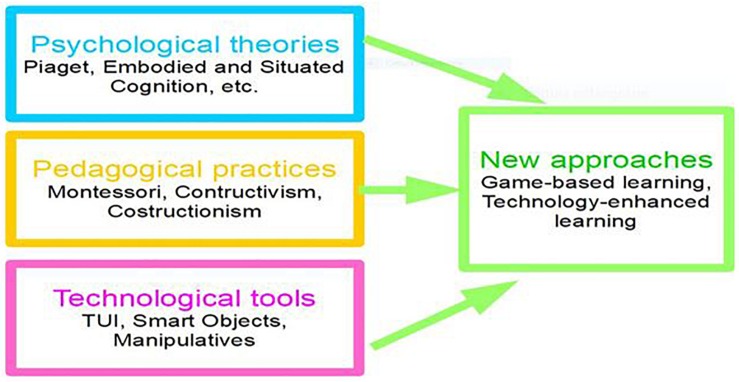
New approaches to foster creativity.

Game-based learning (with digital games) and technology-enhanced learning are two methodological approaches that strongly rely on digital elements, but they do not neglect physical materials. In particular there are some technologies that allow the bridging of these sides (Figure 2).

In this paper we support the use of technological tools in education, but we should be aware that there is also the danger of an excessive or distorted use of technology in educational processes (Desmurget, 2019). In our opinion children should use technology accompanied by someone (an adult or a peer), which promotes and frames the process of knowledge building with technology (Ponticorvo et al., 2018b).

In the next subsection we will introduce some of the processes that can be employed to integrate physical and digital materials.

### Bridging Physical and Digital Materials: TUI and Smart Objects

In recent years, the progress made in the technological field has enabled the possibility of more interfaces. Interfaces allow the joining of two different entities, two separate domains. In the field of information science and what is interesting for the present paper, some interfaces are particularly relevant: the TUI, tangible user interfaces (Blackwell et al., 2007; Ishii, 2008). In this case what is joined is the edge between the digital and physical dimensions so as that a user can interact with information (or data) at the digital level by interacting with the physical environment—for example manipulating tangible objects at a cognitive level (which is very relevant), as we have seen in the previous section.

If we consider the education field, allowing the connection between physical and digital dimensions offers new opportunities under the approach of TEL. It is possible, in fact, to integrate tangible and physical materials traditionally used in education into a new life in the digital universe. This way educational materials such as logic blocks, cards, counting chips, rods, manipulatives in general, etc. (Di Fuccio et al., 2015, 2016; Ferrara et al., 2016; Ponticorvo et al., 2018a, b, 2019) become Smart Objects (Kortuem et al., 2009). A Smart Object can have sensors and processors that, thanks to software, can be in connection and process information together with other objects and with the user.

In sum, Smart Objects are computationally enhanced versions of everyday objects that can also process data, exploit sensors to get information and affect their environment; moreover they can be connected one with the other thanks to the Internet, generating an Internet of Things (IoT). IoT (Li et al., 2015) and Smart Objects technologies have been introduced in the educational domain, offering opportunities and challenges (Domínguez and Ochoa, 2017). Indeed the technological tools can widen the space and time devoted to education, can overcome barriers and open new educational pathways, but this introduction must follow an educational framework that prevents the drawbacks and threats of technology in education (Selwyn, 2016; Collins and Halverson, 2018). Using Tangible User Interfaces (TUI) allows the physical embodiment of digital information, extending the accessibility of objects in the physical world (including tangible materials and active surfaces) which allows the joining together of the digital world and physical objects (see Figure 2).

TUI, Smart objects, and manipulatives represent the technological tools and instruments through which it is possible to put into practice the described approaches of game-based learning and technology-enhanced learning, based on the pedagogical approaches that value the active construction of knowledge and the psychological theories described above that give importance to active interactions with the physical world for framing cognition. This is represented in Figure 2, where it is possible to find a sketch of the connections between these three elements.

In the next section we will describe the application of these principles in a case-study where technology is used to stimulate creativity through its emotional side in a non-verbal participant with autism.

## Fostering Emotion Recognition to Stimulate Creativity by Technology in Children With Autistic Spectrum Disorder (Asd)

As hinted at in the introduction, creativity is connected with the emotional sphere. Emotional competence (Saarni, 1999) refers to the ability to deal effectively with emotions, in terms of understanding others’ emotions, expressing appropriate emotions in a certain context, and regulating them in order to adapt to specific situations. Positive emotional states can increase creativity (Fredrickson et al., 2003), leading to the production of more ideas, even if these ideas aren’t necessarily more original. Also, negative emotions like sadness, anger, and disappointment can help the individual to generate more ideas.

Creativity and emotions are strongly connected in children that have a typical development, as shown by different studies. Fluidity, an aspect that describes the production of a good number of ideas, and flexibility, producing original ideas, appear to have a significant link to emotional competence (Sánchez-Ruiz et al., 2011; Hoffmann and Russ, 2012). If we consider a non-normative population, such as children with Autism Spectrum Disorder, there are some studies that focus on creativity and show that these children can have a high degree of creativity, together with some difficulties related to emotional competence.

Recent studies have shown that for people with ASD video-modeling is particularly effective. Video-modeling is a methodology, integrated with technology, whose functioning is based on learning by observation and imitation. This means that children exposed to video-modeling, starting from the digital material represented by the video, can reproduce what they see with their own bodies, acting on the physical side.

Video-modeling, in more detail, consists in the observation of a video, in which a model shows a target behavior, and the subsequent imitation of that model. A lot of individuals with autism benefit from visually cued instructions (Frith, 2003). Moreover, studies indicated that video-modeling is effective for learning emotional skills: this methodology can support children with autism to recognize emotions and to perceive and respond appropriately to facial expressions (Axe and Evans, 2012; Chen et al., 2016).

Individuals with ASD often present difficulties in the expressive behavior of their emotions, especially in modulating the expression of their face based on the affective state experienced. The expression of emotions is sometimes absent, sometimes ambiguous; in addition, individuals with ASD experience major difficulties in recognizing and interpreting emotional expressions in others. Difficulties in emotion expression and recognition can lead to frustration and dysfunctional behaviors. Furthermore, the poor understanding of one’s emotional state can influence the creative capacities of a child.

### Method

In this paper we describe a methodological approach to fostering creativity acting on the emotional side, by video-modeling. The goal was helping a child with ASD to learn and develop emotional skills, particularly the communication of their own and others’ emotional states, and to verify if the acquisition of emotion recognition was useful for fostering creativity in a drawing task. The chosen methodological design is a multiple baseline across behaviors, A-B-A type, commonly used in ABA treatments. This research design A-B-A belongs to the single-case study where with letter A we intend the baseline condition, whereas the letter B indicates the treatment condition. With an example: A is a situation of anxiety; B is the treatment for reducing anxiety. We identify a real effect of the treatment when the curve undergoes a significant change. The treatment phase starts when the behavior is stable. The target behavior is measured during both phases and results are then compared. Some variants of AB experimental design can foresee a stricter control on variables to have stronger conclusions. With ABA (also called reversal design), in this context, we refer to a research design built on AB that then integrates a follow-up phase after the treatment that includes repeated measures (Horner et al., 2005). The participant was a 6-year-old child with a mild-level condition of Autism Spectrum Disorder.

During the intervention, the following target behaviors were identified:

- To show correctly an emotional state through the change of face expressions; for emotion facial expression, reference was made to the theory by Ekman and Friesen (2003), as it provided useful guidance in experiments on emotion.
- To properly label an emotional state, therefore recognizing and naming an own emotional state.
- To correctly label an emotional state experienced by others, therefore recognizing and naming the emotional state shown by others through facial expression and vocalization.

The categories of emotions selected for the intervention were joy, anger and surprise: these emotions can vary in intensity but are universally recognized and expressed in the same way.

The baseline sessions allowed the tracing of the baseline for the child, whereas the intervention consisted of a video-modeling session.

Data were collected using the traditional procedure of the Momentary Time Sample Recording (MTSR) (Powell et al., 1975) that is to correctly show the emotional state of joy through the change of the face. MTSR is a data-recording technique that is usually used when the observed behavior is not easily quantifiable.

### Results

Results indicated that video-modeling was effective for the acquisition of social and emotional behavior skills in children with ASD, and this led to an improvement of creativity as shown in drawing. The task given to the child consists of recognizing the appropriate emotion and drawing the expression of the mouth to fit the picture, considering all the elements in the face (eyes, eyebrows, nose and signs on cheeks).

**FIGURE 3:**
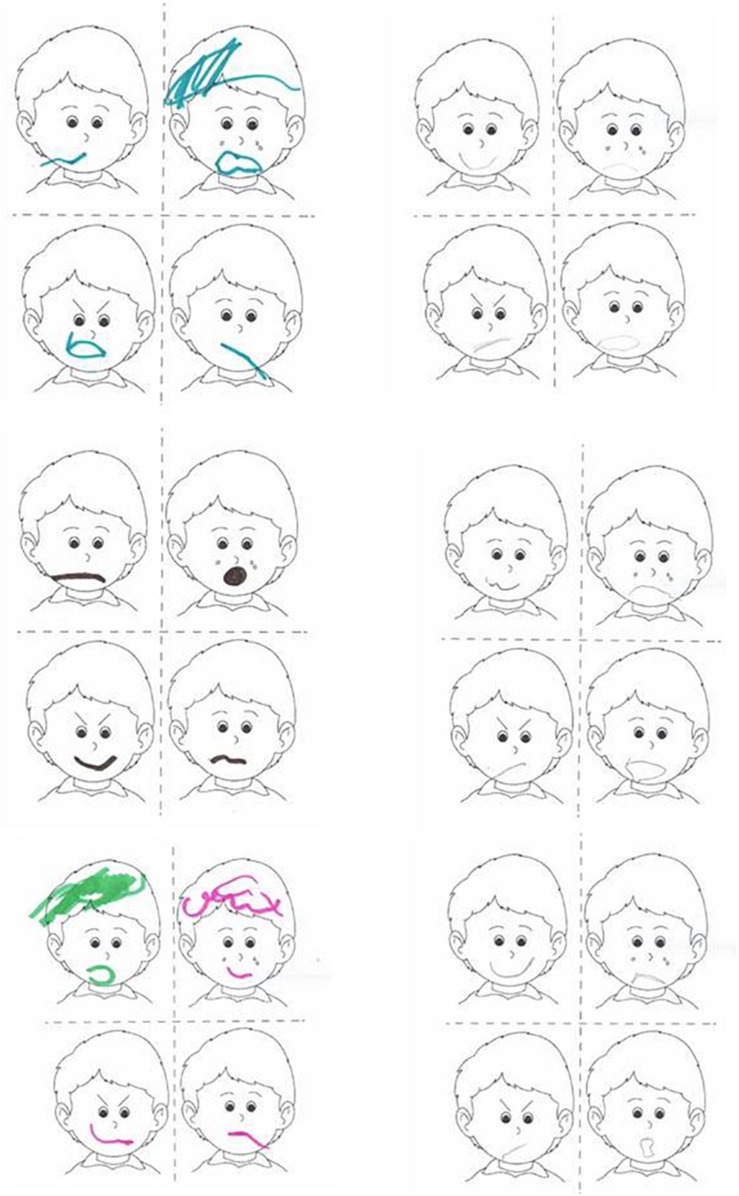
Drawing by the children before **(left)** and after **(right)** the video-modeling intervention.

In fact, as it is possible to observe in Figure 3, compared with the children’s drawing before and after the video-modeling intervention, children were able to recognize the emotions and to reproduce them in a more appropriate and richer way in their productive drawing.

Figure 3 shows the ability to recognize emotions by the child with ASD is ameliorated between BEFORE (on the left) and AFTER (on the right) the treatment with video-modeling. We can observe that discrimination of emotion is better on the right, where the drawn mouth is more coherent with the face elements.

The present study represents a first example of the application of the core ideas of this paper to stimulate creative processes in children with atypical developmental trajectories. It has many limitations that will be overcome with the definition of a research methodology to be applied to a wider group of participants, and also to children with typical development.

## Discussion and Conclusion

Creativity has both a cognitive and emotional side and it is strongly connected with the materials that are employed during creative processes. In this paper we have tried to put together these dimensions to propose methodologies and tools that can be used to stimulate creativity in children. In particular we have shown that educational materials that involve both digital and physical materials, at the edge between these domains, can be particularly effective in having positive effects on creativity mediated by cognitive functions and emotional processes: this happens through the body and the interactions it has with the physical world.

Moreover, we have introduced some useful indications deriving from intervention in a non-normative sample, that is children with ASD. It is also possible to observe in this case that creativity can be improved by stimulating emotional competence and this can be done using materials that bridge digital and physical borders. The reported study is only the starting point of a validation pathway that will cover research on children with typical development, adults and elder people. In fact, these results will be further challenged by widening the sample involved in the experiment, proposing more interventions with different materials and trying this methodology on other groups in different phases of development. In particular, as elderly people can benefit from creativity stimulating intervention, the next step will involve aged people with and without impairments. The experimental design will also be enriched so as to study these wider samples longitudinally and transversally, and experimental protocols will be introduced to have a stronger control over variables.

Creativity can be a resource for everyone in every phase of life: stimulating it with physical and digital materials can be effective. The present study showed an example of how to follow this route, which will be further investigated and developed.

## Data Availability Statement

The datasets generated for this study are available on request to the corresponding author.

## Ethics Statement

Ethical review and approval was not required for the study on human participants in accordance with the local legislation and institutional requirements. Written informed consent to participate in this study was provided by the participants’ legal guardian/next of kin.

## Author Contributions

MP, LS, and OM conceived the original plan of the manuscript. AR designed and carried out the experiment. MP took the lead in writing the manuscript with the support of LS and AR. MP, LS, OM, and AR discussed the manuscript draft and contributed to the submitted manuscript.

## Conflict of Interest

The authors declare that the research was conducted in the absence of any commercial or financial relationships that could be construed as a potential conflict of interest.

## Footnotes

1 https://oxfordre.com/education/view/10.1093/acrefore/9780190264093.001.0001/acrefore-9780190264093-e-383

## References

[B1] Adams-Price C. E. Nadorff D. K. Morse L. W. Davis K. T. Stearns M. A. (2018). The creative benefits scale: connecting generativity to life satisfaction. *Int. J. Aging Hum. Dev.* 86 242–265. 10.1177/0091415017699939 28351155

[B2] Amabile T. M. (1996). *Creativity and Innovation in Organizations.* Cambridge, MA: Harvard Business School.

[B3] Axe J. B. Evans C. J. (2012). Using video modeling to teach children with PDD-NOS to respond to facial expressions. *Res. Autism Spectr. Disord.* 6 1176–1185.

[B4] Beghetto R. A. (2013). *Killing Ideas Softly: The Promise and Perils of Creativity in the Classroom.* Charlotte: IAP Information Age Publishing.

[B5] Beghetto R. A. Kaufman J. C. (2007). Toward a broader conception of creativity: a case for “mini-c” creativity. *Psychol. Aesthet. Creat. Arts* 1 73–79.

[B6] Beghetto R. A. Kaufman J. C. Baer J. (2014). *Teaching for Creativity in the Common Core Classroom.* New York, NY: Teachers College Press.

[B7] Blackwell A. F. Fitzmaurice G. Holmquist L. E. Ishii H. Ullmer B. (2007). “Tangible user interfaces in context and theory,” in *CHI’07 Extended Abstracts on Human Factors in Computing Systems*, eds Rossom M. B. Gilmore D. (New York, NY: ACM Association for Computing Machinery), 2817–2820.

[B8] Bruner J. S. (1961). *The Act of Discovery*, Vol. 31 Cambridge, MA: Harvard Educational Review, 21–32.

[B9] Chen C. H. Lee I. J. Lin L. Y. (2016). Augmented reality-based video-modeling storybook of nonverbal facial cues for children with autism spectrum disorder to improve their perceptions and judgments of facial expressions and emotions. *Comput. Hum. Behav.* 55 477–485.

[B10] Clancey W. J. (1995). “A tutorial on situated learning,” in *Proceedings of the International Conference on Computer and Education*, ed. Self J. (Charlottesville, VA: AACE), 49–70.

[B11] Clark A. (1998). *Being there: Putting Brain, Body, and World Together Again.* Cambridge, MA: MIT press.

[B12] Cohen G. (2006). Research on creativity and aging: the positive impact of the arts on health and illness. *Generations* 30 7–15.

[B13] Collins A. Halverson R. (2018). *Rethinking Education in the Age of Technology: The Digital Revolution and Schooling in America.* New York, NY: Teachers College Press.

[B14] Craft A. Cremin T. Hay P. Clack J. (2014). Creative primary schools: developing and maintaining pedagogy for creativity. *Ethnogr. Educ.* 9 16–34.

[B15] Cremin T. Chappell K. Craft A. (2013). Reciprocity between narrative, questioning and imagination in the early and primary years: examining the role of narrative in possibility thinking. *Think. Skills Creat*. 9, 135–151. 10.1016/j.tsc.2012.11.003

[B16] Csikszentmihalyi M. (1996). *Creativity: The Work and Lives of 91 Eminent People.* New York, NY: Harper Collins.

[B17] Dell’Aquila E. Marocco D. Ponticorvo M. Di Ferdinando A. Schembri M. Miglino O. (2016). *Educational Games for Soft-Skills Training in Digital Environments: New Perspectives.* Cham: Springer International Publishing Switzerland.

[B18] Desmurget M. (2019). *La Fabrique du Crétin Digital-Les Dangers des Écrans Pour nos Enfants.* Paris: Le Seuil.

[B19] Di Dio C. Macaluso E. Rizzolatti G. (2007). The golden beauty: brain response to classical and renaissance sculptures. *PLoS One* 2:e1201 10.1371/journal.pone.0001201 PMC2065898 18030335

[B20] Di Fuccio R. Ponticorvo M. Di Ferdinando A. Miglino O. (2015). “Towards hyper activity books for children. connecting activity books and montessori-like educational materials,” in *Design for Teaching and Learning in a Networked World*, eds Conole G. Klobučar T. Rensing C. Lavoué J. E. (Cham: Springer), 401–406.

[B21] Di Fuccio R. Ponticorvo M. Ferrara F. Miglino O. (2016). “Digital and multisensory storytelling: narration with smell, taste and touch,” in *European Conference on Technology Enhanced Learning*, eds Verbert K. Sharples M. Klobucar T. (Cham: Springer), 509–512.

[B22] Dietrich A. Kanso R. (2010). A review of EEG, ERP, and neuroimaging studies of creativity and insight. *Psychol. Bull.* 136 822–848. 10.1037/a0019749 20804237

[B23] Domínguez F. Ochoa X. (2017). “Smart objects in education: an early survey to assess opportunities and challenges,” in *Proceedings of the 2017 Fourth International Conference on eDemocracy & eGovernment (ICEDEG)*, (Quito: IEEE), 216–220.

[B24] Ekman P. Friesen W. V. (2003). *Unmasking the Face: A Guide to Recognizing Emotions from Facial Clues.* Los Altos, CA: ISHK Institute for the Study of Human Knowledge.

[B25] Ferrara F. Ponticorvo M. Di Ferdinando A. Miglino O. (2016). “Tangible interfaces for cognitive assessment and training in children: LogicART,” in *Smart Education and e-Learning 2016*, eds Uskov V. Howlett R. J. Jain Lakhmi C. (Cham: Springer), 329–338.

[B26] Fredrickson B. L. Tugade M. M. Waugh C. E. Larkin G. R. (2003). What good are positive emotions in crisis? A prospective study of resilience and emotions following the terrorist attacks on the United States on September 11th, 2001. *J. Pers. Soc. Psychol.* 84 365–376. 10.1037//0022-3514.84.2.365 12585810 PMC2755263

[B27] Frith U. (2003). *Autism: Explaining the Enigma.* Hoboken NJ: Blackwell Publishing.

[B28] Gajda A. Karwowski M. Beghetto R. A. (2017). Creativity and academic achievement: a meta-analysis. *J. Educ. Psychol.* 109 269–299.

[B29] Galperin P. I. (1969). “Stages in the development of mental acts,” in *A Handbook of Contemporary Soviet Psychology*, eds Cole M. Maltzman I. (New York, NY: Basic Books), 249–273.

[B30] Galperin P. I. (1989). Mental actions as a basis for the formation of thoughts and images. *Sov. Psychol.* 27 45–64.

[B31] Giglio M. Cruz-Ortiz J. (2015). *Creative Collaboration in Teaching.* New York, NY: Palgrave Macmillan.

[B32] Glãveanu V. P. (2013). Rewriting the language of creativity: the five A’s framework. *Rev. Gen. Psychol.* 17 69–81.

[B33] Goodyear P. Retalis S. (2010). *Technology-Enhanced Learning.* Rotterdam: Sense Publishers.

[B34] Harris A. de Bruin L. R. (2018). Secondary school creativity, teacher practice and STEAM education: an international study. *J. Educ. Change* 19 153–179.

[B35] Hetzroni O. Agada H. Leikin M. (2019). Creativity in autism: an examination of general and mathematical creative thinking among children with autism spectrum disorder and children with typical development. *J. Autism Dev. Disord.* 49 3833–3844. 10.1007/s10803-019-04094-x 31183666

[B36] Hoffmann J. Russ S. (2012). Pretend play, creativity, and emotion regulation in children. *Psychol. Aesthet. Creat. Arts* 6 175–184.

[B37] Horner R. H. Carr E. G. Halle J. McGee G. Odom S. Wolery M. (2005). The use of single-subject research to identify evidence-based practice in special education. *Except. Child.* 71 165–179. 10.1044/0161-1461(2009/08-0128) 20421615

[B38] Houtz J. C. Krug D. (1995a). Assessment of creativity: resolving a mid-life crisis. *Educ. Psychol. Rev.* 7 269–300.

[B39] Houtz J. C. Krug D. (1995b). Preface to special issue on the educational psychology of creativity: toward an educational psychology of creativity? *Educ. Psychol. Rev.* 7 137–139.

[B40] Ishii H. (2008). The tangible user interface and its evolution. *Commun. ACM* 51 32–36.

[B41] Johnson C. Wilbrecht L. (2011). Juvenile mice show greater flexibility in multiple choice reversal learning than adults. *Dev. Cogn. Neurosci.* 1 540–551. 10.1016/j.dcn.2011.05.008 21949556 PMC3177141

[B42] Jonassen D. H. (1994). Technology as cognitive tools: learners as designers. *ITForum Pap.* 1 67–80.

[B43] Jonassen D. H. Howland J. Marra R. Crismond D. (2008). *Meaningful Learning with Technology.* Upper Saddle River, NJ: Pearson.

[B44] Kapp K. M. (2012). *The Gamification of Learning and Instruction.* San Francisco, CA: Wiley.

[B45] Karwowski M. Gralewski J. Szumski G. (2015). Teachers’ effect on students’ creative self-beliefs is moderated by students’ gender. *Learn. Individ. Differ.* 44 1–8.

[B46] Kaufman J. C. Beghetto R. A. (2009). Beyond big and little: the four c model of creativity. *Rev. Gen. Psychol.* 13 1–12.

[B47] Kleibeuker S. W. De Dreu C. K. Crone E. A. (2013). The development of creative cognition across adolescence: distinct trajectories for insight and divergent thinking. *Dev. Sci.* 16 2–12. 10.1111/j.1467-7687.2012.01176.x 23278922

[B48] Kortuem G. Kawsar F. Sundramoorthy V. Fitton D. (2009). Smart objects as building blocks for the internet of things. *IEEE Internet Comput.* 14 44–51.

[B49] Li S. Da Xu L. Zhao S. (2015). The internet of things: a survey. *Inf. Syst. Front.* 17 243–259.

[B50] McCormick D. J. Plugge C. D. (1997). “If I am an artist, what’s wrong with my picture? Rediscovering your creativity in a grown-up world,” in *Proceedings of the Annual AEE International Conference Deeply Rooted, Branching’Out, 1972-1997*, Asheville.

[B51] Montessori M. (1995). *The Absorbent Mind.* New York, NY: Henry Holt and Company Inc.

[B52] Montessori M. (2013). *The Montessori Method.* Piscataway, NJ: Transaction publishers.

[B53] Ogoemeka O. H. (2011). Emotional intelligence and creativity in teacher education. *Int. J. Soc. Sci. Educ.* 3 124–129.

[B54] Papert S. (1980). *Mindstorms: Children, Computers, and Powerful Ideas.* New York, NY: Basic Books.

[B55] Papert S. Harel I. (1991). Situating constructionism. *Constructionism* 36 1–11. 10.1177/1049732313488837 23656723 PMC3680787

[B56] Piaget J. (1952). *The Origins of Intelligence in Children.* New York, NY: International Universities Press.

[B57] Piaget J. (1964). Part I: cognitive development in children: Piaget development and learning. *J. Res. Sci. Teach.* 2 176–186.

[B58] Ponticorvo M. Dell’Aquila E. Marocco D. Miglino O. (2019). Situated psychological agents: a methodology for educational games. *Appl. Sci.* 9:4887.

[B59] Ponticorvo M. Di Fuccio R. Ferrara F. Rega A. Miglino O. (2018a). “Multisensory educational materials: five senses to learn,” in *Proceedings of the International Conference in Methodologies and Intelligent Systems for Technology Enhanced Learning*, eds Mascio Di T. Vittorini P. Gennari R. De la Prieta F. Rodríguez S. Temperini M. (Cham: Springer), 45–52.

[B60] Ponticorvo M. Rega A. Miglino O. (2018b). “Toward tutoring systems inspired by applied behavioral analysis,” in *Proceedings of the International Conference on Intelligent Tutoring Systems*, eds Nkambou R. Azevedo R. Vassileva J. (Cham: Springer), 160–169.

[B61] Powell J. Martindale A. Kulp S. (1975). An evaluation of time-sample measured of behaviour 1. *J. Appl. Behav. Anal.* 8 463–469. 16795509 10.1901/jaba.1975.8-463 PMC1311883

[B62] Rambusch J. (2006). “Situated learning and Galperin’s notion of object-oriented activity,” in *Proceedings of the Annual Meeting of the Cognitive Science Society*, Skövde.

[B63] Rambusch J. Ziemke T. (2005). “The role of embodiment in situated learning,” in *Proceedings of the 27th Annual Conference of the Cognitive Science Society*, (Mahwah, NJ: Lawrence Erlbaum), 1803–1808.

[B64] Rhodes M. (1961). An analysis of creativity. *Phi Delta Kappa* 42 305–310.

[B65] Rhodes M. (1987). “An analysis of creativity,” in *Frontiers of Creativity Research: Beyond the Basics*, ed. Isaksen S. (Buffalo, NY: Bearly Ltd), 216–222.

[B66] Roth W. M. (2002). From action to discourse: the bridging function of gestures. *Cogn. Syst. Res.* 3 535–554.

[B67] Runco M. A. Jaeger G. J. (2012). The standard definition of creativity. *Creat. Res. J.* 24 92–96.

[B68] Saarni C. (1999). *The Development of Emotional Competence.* New York, NY: Guilford Press.

[B69] Sánchez-Ruiz M. J. Hernández-Torrano D. Pérez-González J. C. Batey M. Petrides K. V. (2011). The relationship between trait emotional intelligence and creativity across subject domains. *Motiv. Emot.* 35 461–473.

[B70] Selwyn N. (2016). *Education and Technology: Key Issues and Debates.* London: Bloomsbury Publishing.

[B71] Shapiro L. (2019). *Embodied Cognition.* Abingdon: Routledge.

[B72] Simonton D. K. (1999). *Origins of Genius: Darwinian Perspectives on Creativity.* Oxford: Oxford University-Press.

[B73] Smith L. Gasser M. (2005). The development of embodied cognition: six lessons from babies. *Artif. Life* 11 13–29. 10.1162/1064546053278973 15811218

[B74] Stanciu M. M. (2015). Embodied creativity: a critical analysis of an underdeveloped. *Procedia Soc. Behav. Sci.* 187 312–317.

[B75] Sternberg R. J. Lubart T. I. (1996). Investing in creativity. *Am. Psychol.* 51 677–688.

[B76] Tobias S. Fletcher J. D. Wind A. P. (2014). “Game-based learning,” in *Handbook of Research on Educational Communications and Technology*, eds Spector J. M. Merrill M. D. Elen J. Bishop M. J. (New York, NY: Springer), 485–503.

[B77] Treffinger D. J. Isaksen S. G. Firestien R. L. (1983). Theoretical perspectives on creative learning and its facilitation: an overview. *J. Creat. Behav.* 17 9–17.

[B78] Vygotsky L. (1978). Interaction between learning and development. *Read. Dev. Child.* 23 34–41.

[B79] Vygotsky L. S. (1967). Play and its role in the mental development of the child. *Sov. Psychol.* 5 6–18.

[B80] Williams F. (1994). *TCD. Test Della Creatività e del Pensiero Divergente.* Trento: Centro Studi Erickson.

